# Technological Characterization of PET—Polyethylene Terephthalate—Added Soil-Cement Bricks

**DOI:** 10.3390/ma14175035

**Published:** 2021-09-03

**Authors:** Tulane Rodrigues da Silva, Daiane Cecchin, Afonso Rangel Garcez de Azevedo, Izabella Valadão, Jonas Alexandre, Flavio Castro da Silva, Markssuel Teixeira Marvila, Murali Gunasekaran, Fabio Garcia Filho, Sergio Neves Monteiro

**Affiliations:** 1 LAMAV—Advanced Materials Laboratory, UENF—State University of the Northern Rio de Janeiro, Av. Alberto Lamego, 2000, Campos dos Goytacazes 28013-602, Brazil; tulanerodrigues@gmail.com (T.R.d.S.); m.marvila@ucam-campos.br (M.T.M.); 2 Department of Agricultural and Environmental Engineering, Federal Fluminense University—UFF, R. Passo da Pátria, 156, Niterói 24210-240, Brazil; daianececchin@id.uff.br (D.C.); flaviocastro@id.uff.br (F.C.d.S.); 3 LECIV—Civil Engineering Laboratory, UENF—State University of the Northern Rio de Janeiro, Av. Alberto Lamego, 2000, Campos dos Goytacazes 28013-602, Brazil; jonasuenf@gmail.com; 4 Department of Civil Engineering, Federal Fluminense University—UFF, R. Passo da Pátria, 156, Niterói 24210-240, Brazil; izabellavaladao@id.uff.br; 5 School of Civil Engineering, SASTRA Deemed University, Thanjavur 613401, India; murali@civil.sastra.edu; 6 Military Engineering Institute, IME—Materials Science Program, Praça Gen. Tibúrcio, 80, Urca, Rio de Janeiro 22290-270, Brazil; fabiogarciafilho@gmail.com (F.G.F.); snevesmonteiro@gmail.com (S.N.M.)

**Keywords:** building materials, PET, compressive strength, water absorption

## Abstract

The ever-growing consumption and improper disposal of non-biodegradable plastic wastes is bringing worrisome perspectives on the lack of suitable environmentally correct solutions. Consequently, an increasing interest in the circular economy and sustainable techniques is being raised regarding the management of these wastes. The present work proposes an eco-friendly solution for the huge amount of discarded polyethylene terephthalate (PET) wastes by addition into soil-cement bricks. Room temperature molded 300 × 150 × 70 mm bricks were fabricated with mixtures of clay soil and ordinary Portland cement added with up to 30 wt.% of PET waste particles. Granulometric analysis of soil indicated it as sandy and adequate for brick fabrication. As for the PET particles, they can be considered non-plastic and sandy. The Atterberg consistency limits indicated that addition of 20 wt.% PET waste gives the highest plasticity limit of 17.3%; moreover, with PET waste addition there was an increase in the optimum moisture content for the compaction and decrease in specific weight. Standard tests showed an increase in compressive strength from 0.83 MPa for the plain soil-cement to 1.80 MPa for the 20 wt.% PET-added bricks. As for water absorption, all bricks displayed values between 15% and 16% that attended the standards and might be considered an alternative for non-structural applications, such as wall closures in building construction.

## 1. Introduction

The scarcity of natural resources and the generation of solid wastes without adequate disposal is of worldwide concern, which makes their reuse feasible in the civil construction sector, besides encouraging sustainable development and a circular economy [1,2,3,4]. The solid waste problem is of concern mainly in urbanized regions and developing countries where collection and disposal services have difficulties dealing with increasing amounts of waste [2]. As a result, waste is either disposed in open, uncontrolled dumps, accounting for 61% of the landfill sector’s CO_2_ emissions, or burned in the open dumps, accounting for 40% of global waste [5]. Sustainable solid waste management has become a necessity for industries seeking to promote industrialization and sustainable development. Government regulations have become more stringent around the world, representing an accelerating factor in the adoption of reverse logistics initiatives, including in countries that have been facing difficulties in recycling processes, thus giving more space for the use of this waste within its life cycle [4].

Global plastic production is growing rapidly and by 2030 the world may produce about 619 million tons of plastic per year [6]. A study by Spósito et al. [7] emphasizes that post-consumer polyethylene terephthalate (PET) products have generated a growing interest regarding their recycling potential and their negative impacts on the environment, such as pollution and long degradation time. According to WWF [8], phasing out single-use plastics has the potential to reduce plastic demand by up to 40% by the year 2030. Thus, the current scenario requires a more sustainable route for the recycling of this waste, which if not realized will increase the environmental imbalance due to its non-biodegradability in nature [8,9].

**Figure 1:**
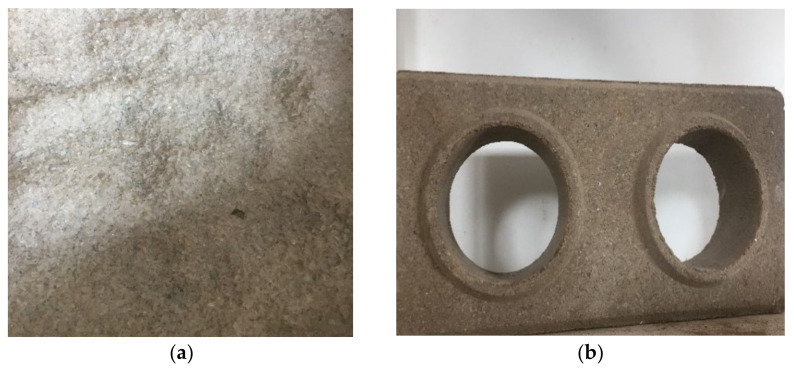
Materials used: (**a**) PET waste; (**b**) soil-cement brick.

The growing demand for sustainable products has encouraged several studies that search for alternative techniques regarding the reuse of waste in construction materials, such as mortars with sugarcane bagasse [10], cement pastes with açai fiber [11,12], blast furnace slag [13,14], construction and demolition waste [15], ceramic materials with rice ash water treatment plant sludge [16], pulp and paper industry sludge [17], construction and demolition waste [18], and agricultural waste [19], as well as concrete with plastic waste [20]. Therefore, research also highlights the reuse of plastic waste in construction materials [19,20] such as paving [21], mortar [7], concrete [19,20,22,23], fired clay blocks, and bricks [24], as well as unfired blocks and bricks [25,26], thus showing PET (Figure 1a) as an addition in the production of these materials.

Among the various building materials available for waste addition stand soil-cement bricks (Figure 1b), the use of soil-cement bricks presents many advantages from the environmental point of view. There is no need for a burning process, which is associated with a reduction of greenhouse gas emissions and enhanced technological properties. Another advantage is the reduction of costs when these bricks are used for the execution of masonry due to the dismissal of the use of mortar to join the bricks of a fitting type [16,27]. Thus, this construction material has potential for use in small and medium sized buildings without structural function, in addition to having a low financial cost [28,29].

The soil-cement bricks allow the incorporation of waste in their composition and reduce costs up to 40% compared to traditional masonry, especially in low-income housing. In this way, the brick can be considered more sustainable in relation to the traditional brick [30]. In this context, it is also possible to verify the use of waste in soil-cement bricks as shown França et al. [28], who studied the durability of soil-cement bricks with incorporation limestone waste. The authors used 30%, 40% and 50% of waste for the manufactured soil-cement mixtures. The results verified that the incorporation of waste rock was technologically feasible. The parameters studied for compressive strength, water absorption and durability showed superior performance of bricks with waste incorporation. Reis et al. [31] evaluated the incorporation of quartzite mining tailings in soil-cement bricks. The authors tested additions of quartzite tailings at 0%, 15% and 30%. The results showed that the compressive strength of the soil-cement bricks decreased with the addition of quartzite waste. However, the authors observed that the results of compressive strength and water absorption performed at 7 and 28 days demonstrated the possibility of using the waste without compromising the physical and mechanical properties required by the standard, thus verifying that the soil-cement brick is a viable technique for the disposal of this type of waste. Kongkajun et al. [32] evaluated soil-cement bricks with incorporation of construction waste (clay bricks) and fiber-cement industry sludge. The authors used 15 wt.% of Portland cement, 15 wt.% of sand and 70 wt.% of laterite to produce the bricks. The clay brick waste was added from 10% to 50 wt.% of laterite in the control samples. Sludge, on the other hand, was added at 5% and 10 wt.% to replace the total weight of the mixture in the control samples. The maximum obtainable substitution of laterite for clay brick waste was 50 wt.% in the mixture. Maximum compressive strength was achieved for the 10% replacement of laterite with clay bricks. Partial replacement of laterite with clay bricks improved the compressive strength of soil-cement bricks for load-bearing brick application. Although the incorporation of silt caused a reduction in the compressive strength of the brick samples compared to the samples prepared from the control sample, they still exceeded the Thailand community product standard. Increasing the percentage of sludge from 0% to 10 wt.% resulted in a significant decrease in thermal conductivity of 45% compared to the control formula. When using the sludge and clay bricks, the thermal conductivity and density of the bricks were further reduced, while their compressive strength and water absorption values were still satisfactory. 

Kouamé et al. [33] verified the influence of shea butter wastes on the physical properties of cement-stabilized soil bricks. The authors used three local clay raw materials consisting mainly of kaolinite, quartz and micaceous phases, as well as shea butter waste and cement. In the mixtures tested, the amount of cement was kept constant (5%), while the amount of shea butter waste varied from 2% to 10%, replacing the soil. The results obtained by the authors showed that the presence of pores due to the shea butter waste influences the reduction of density and thermal conductivity of the bricks. A 25% decrease in thermal conductivity was verified for the samples with clay F, 16% for the samples with clay K, and 22% with clay Y. The authors concluded that the bricks showed good stiffness related to the presence of cementation phases. Therefore, for the samples with clay F and clay Y, the replacement rate of 6% by the shea butter waste was sufficient as compared to 8% for the formulations with K to obtain a physical property. Thus, they found that the shea butter waste offered good thermal insulation and good stiffness properties with a lower amount of cement used. 

Vilela et al. [34] evaluated the incorporation of mining waste in soil-cement bricks for soil substitution at 10%, 20%, 30% and 40% of waste. As for the mechanical strength, the authors found that all treatments showed values above the required standards, with the minimum standard value being set at 2.0 MPa. The treatment with 10% mining waste presented the best results. The thermal conductivity showed a direct link with the density of the bricks since the increase in density (bricks with 40% mining waste) led to a material with a lower heat dissipation property. The study showed that the addition of mining waste to soil-cement bricks met all the required standards [34].

In this sense, the use of plastic waste can also be considered in studies such as [7], which showed that hydrated mortars produced with PET bottle waste replaced the fine aggregate in the mixture and suffered changes in properties in their fresh and hardened states. Reference [21] investigated the effects of PET waste on hardened properties in high strength concrete and the investigation showed the interference of high temperatures on concrete properties, in this case, the occurrence of material fragmentation and the release of greenhouse gases. Reference [22] evaluated PET blends for sidewalk sub-bases, highlighting bottles and food packaging taken from collection points and crushed into mixtures with two main constituents of waste materials or construction and demolition by-products: concrete aggregate and crushed brick.

Studies by [24] found that compared to normal concrete, high strength concrete has a failure mode and that the lack of ductility can be solved by using different types of plastic fibers. Akinyele et al. (2020) evaluated the incorporation of PET into fired blocks varied by 0%, 5%, 10%, 15% and 20% and found changes in the samples with respect to high temperature, compressive strength and water absorption. Reference [23] investigated concrete with added waste plastic by varying it at 0%, 5%, 10%, 15% and 20% and found that the concrete showed failure in shear, while in the hardened state it showed gradual reduction in the strength of the material as more granular plastic was added to the concrete mix.

According to [27], the addition of plastic waste in pressed blocks with a variation of 0%, 1%, 3%, and 7% showed that the compressive strength of the stabilized earth block without additives was low and that there was an initial increase in compressive strength with increasing plastic waste. The optimum compressive strength for the study was obtained for blocks containing 1% crushed plastic waste, whose particle sizes were less than 6.3 mm. The increase in compressive strength was 244.4% when compared to the block without the addition of plastic waste.

This paper aimed to evaluate the influence of the incorporation of polyethylene terephthalate (PET) waste in the properties of soil-cement bricks. The study emphasizes mainly the analysis of characterization of the materials used, since this type of brick has particularities for its manufacturing. Therefore, characterization of the soil was performed, as well as the PET waste, to see the relationship of both in the mixtures. The compaction curves were also highlighted, since most studies have difficulties regarding the optimum moisture content used in the production of mixtures for this type of masonry. Moreover, this work also presents the PET waste as an innovation since it is still minimally used and not often discussed in research on the subject of soil-cement.

## 2. Materials and Methods

### 2.1. Materials and Mixtures

The sandy-clay soil used in the experiment was collected from a deposit located in Campos dos Goytacazes, Rio de Janeiro, Brazil, at a depth of 1.0 m on average, after removing the top-soil layer with a high percentage of organic matter. The samples were separated in plastic bags and kept hermetically sealed in order to maintain the humidity of the material. After collection, the soil was divided into different portions for homogeneity of the moisture of the samples. Then, the soil was crushed to reduce its volume, thus standardizing its granulometry. After this step, the soil was sieved, using a sieve with an aperture of 4.75 mm according to [35]. The distribution of the grain sizes of the collected soil samples and mixtures was performed by sieving, according to the procedures by [36]. The consistency limits (Atterberg) of the soil and mixtures were tested, determining the plastic properties of the samples according to [36,37]. Normal Proctor compaction tests were performed for soil and mixtures according to [38,39].

The PET waste used in this research presented particles of virgin material supplied by industries, with physical breakdowns and without contaminants as well as recycled PET treated post-consumer waste, i.e., mixed, with a uniform granulated aspect and white color. As a soil stabilizer, ordinary Portland cement type CPV-ARI was used, which has a high initial strength and is widely used in materials that require rapid hardening [16,27]. By considering that the bricks produced are molded, the use of this type of cement facilitates the transportation of the machine to the curing sites, avoiding its disintegration.

To perform the morphological analysis of the PET incorporated into the bricks, scanning electron microscopy (SEM) model FEG Quanta 250, belonging to the Laboratory of Electron Microscopy (LME) of the Military Institute of Engineering, Rio de Janeiro, Brazil (IME) was used. For this, the PET sample was coated with gold film and analyzed at 60 × magnification. To make the bricks, the mixture used was in the proportion of 1 part cement to 6 parts of soil (1:6). The bricks with 10% addition of PET in relation to the soil, 20% addition of PET in relation to the soil and 30% addition of PET in relation to the soil were analyzed in addition to the reference mixture, which was without any addition of waste (Table 1).

Before manufacturing, the materials used in each mixture were weighed with the aid of a digital scale. After weighing, the mixture (soil, waste and cement) was homogenized with the aid of a mechanical mixer. The addition of water was done with a sprayer in order to distribute the water in the mixture in a controlled and uniform way, thus avoiding the formation of lumps of soil concentrated by the excess of water. The amount of water used was calculated according to the optimum moisture found in the Normal Proctor compaction tests, as shown in studies by [27,33].

### 2.2. Experimental Procedures

Compaction tests were performed for the soil and the mixture with PET according to [37,38]. They related the moisture content to be used with the specific weight of the soil samples and the mixtures with PET. To perform the compaction test, a cylindrical mold (Normal Proctor) was used, with a base attached to a metal socket which has a drop control apparatus [40,41].

The molding process of the bricks was performed according to [35] at the company Arte Cerâmica Sardinha, located in Campos dos Goytacazes. A hydraulic press, Model 7000 Turbo II, of the manufacturer Ecomáquinas, Navegantes, Santa Catarina, Brazil, was used, which has a molding capacity of up to 2 bricks at a time and a compression pressure of approximately 15 tons. The bricks were made in the size of 30 × 15 cm (length × width) and variable height of 7 cm ± 1 cm, with 2 holes and a useful area of 80%.

**Figure 2:**
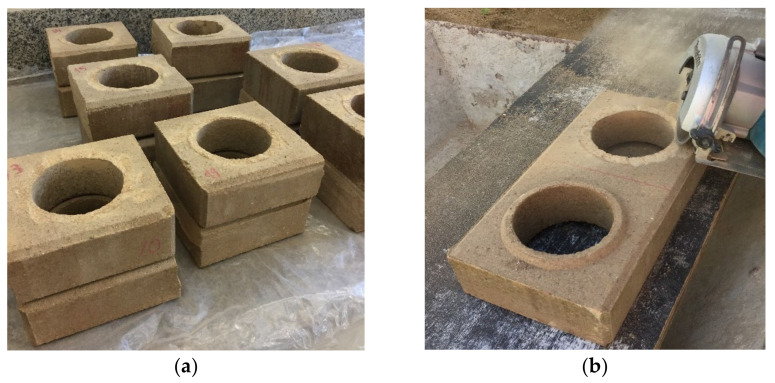
Brick capping step: (**a**) Bricks cut in half; (**b**) upper part of socket removed.

After making the bricks, the curing process was carried out by water sprinkling for 28 days. The process occurred in a humid chamber, where the specimens were placed on pallet racks covered with plastic sheeting, and during the 28 days constant humidity was added to the bricks through the water pump sprayer to help in the hydration process of the cement. The compressive strength was analyzed after 7 and 28 days of curing. For the statistical analysis, 7 samples of each treatment were used. The dimensions of the samples were measured with a calliper, according to the ABNT standards [40,41]. Then the bricks were cut in half (Figure 2a) in the transverse direction, having the upper part of the socket removed, as recommended by the ABNT standard [41]. The cuts were made with the aid of an electric saw (Figure 2b). Next, the cut halves were overlapped and joined by cement paste and then the capping was performed.

The cement used to make the capping paste was the same CPV-ARI used in the manufacture of the bricks. After capping, the bricks were submerged in water for 24 h to ensure their saturation before rupture, which was performed in the press Model 100 T Manual Hydraulic Press DIG.110/220, Solotest, São Paulo, Brazil. 

The water absorption test of the bricks was performed according to the ABNT standard [42,43] at 28 days after manufacturing. Three bricks of each mixture were used, which were dried in a SOLAB oven, SL-100 model, with temperatures ranging between 105 °C and 110 °C until reaching constant mass, for 48 h and then stored. After this period, the weight of each of the bricks was measured with the aid of a digital balance brand Marte, Model AD5002, São Paulo, Brazil to obtain the dry mass (g), as recommended by the ABNT standard [43]. Then, the bricks were immersed in water for 24 h, and after this saturation step, they were dried superficially with the help of a clean and dry cloth and then weighed again to obtain the wet mass (g). The water absorption of the bricks was obtained, according to the ABNT standard [43]:

(1) $$A\left(\%\right)=\frac{\left(m1-m2\right)}{m2}\times 100\;$$

where: A—is the water absorption (%); m1—is the dry mass of the specimen (g); m2—is the mass of the saturated specimen (g).

Analysis of Variance (ANOVA) was used to verify the existence of significant differences between the results obtained. Statistical differences were confirmed by means of the comparison of means test, using Tukey’s method (*p* < 0.05). The experimental design used was the Completely Randomized Design for the two variables analyzed: water absorption and compressive strength. For water absorption, three specimens were used for each treatment (28 days of curing): 0%, 10%, 20% and 30%. For compressive strength, seven specimens were used for each of the four treatments (7 days and 28 days of curing). 

## 3. Results and Discussion

### 3.1. Characterization of Materials and Mixtures

#### 3.1.1. Granulometric Analysis

**Figure 3:**
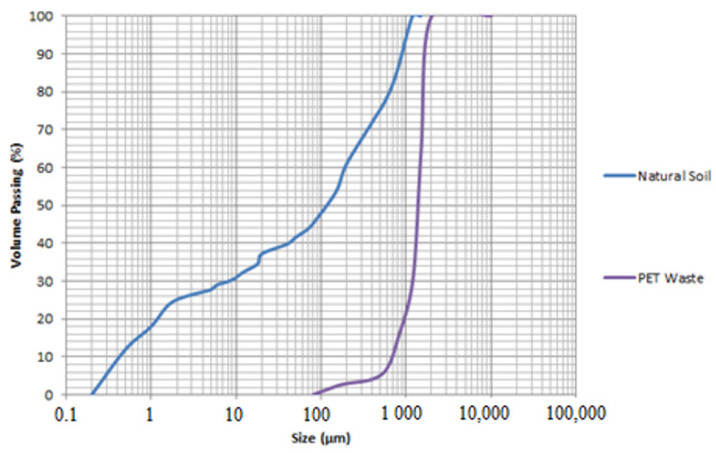
Natural soil and PET waste granulometric analysis.

The soil used for making the bricks, according to its particle distribution, showed 57.1% sand, 24.3% clay and 18.6% silt fraction, which is classified as sandy clay soil according to the ABNT standard [44]. This soil is considered suitable for making bricks as recommended by [45], who state that soils with sandy characteristics are the most suitable for the confection of soil-cement bricks, and also by the ABCP [46]. Indeed, the results of the granulometric analysis of the soil indicated that 100% passed through the ABNT 4.8 mm sieve (n°. 4) and 10% to 50% passed through the ABNT 0.075 mm sieve (n° 200), as observed in the granulometric curve presented in Figure 3. 

The particle size distribution of PET particles was 99.9% sandy, 85.5% coarse, 11.5% medium and 2.9% fine particles, as shown in Figure 3. Since PET did not present consistency limits nor hygroscopicity, it can be considered non-plastic and sandy, according to the ABNT standard [44].

#### 3.1.2. Consistency Limits

The consistency limits of the soil used presented the plasticity index (PI) values in Table 2. Thus, it was possible to verify that the values were adequate, since the PI for the production of soil-cement bricks should be up to 18%, as shown in studies [45,46]. In addition, the results reached the reference values for the manufacture of soil-cement bricks, i.e., soils with the maximum limit of 45% for liquidity limit (LL) and 18% for plasticity limit (PL), as well as those recommended by the ABNT standard [35].

Differently from the results of the soil (reference sample), the values of the PET-Soil cement mixtures showed decreased LL, PL and PI compared to natural soil. This occurred due to the addition of PET waste (sandy fraction), showing that with an increase in the percentage of addition, the greater the amount of water needed in the mixture to get out of its plastic state. This can be related to the workability of the mixture, i.e., the greater the amount of water added, the lower the workability of the mixture [47,48].

#### 3.1.3. Moisture Content and Compaction Energy

The optimum compaction moisture results with the respective maximum densities of the mixtures are presented in Table 3.

**Figure 4:**
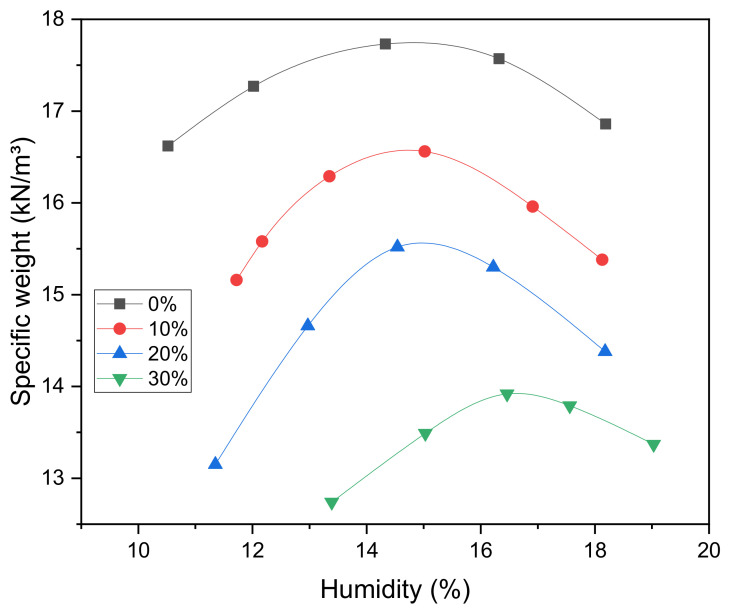
Compaction curves and optimum moisture of mixtures. Percentages (%) indicate the amount of water content in each mixture (0%, 10%, 20% and 30%).

The results in this table showed that with the increase of PET waste, there was an increase in the optimum moisture content of compaction and a decrease in the maximum specific weight. As for the decrease in maximum specific weight, this is due to the density value of the PET waste being lower than that of the natural soil. Moreover, the sample mass decreased as water was added to the compaction process because the waste did not have plastic properties. With this, as the water addition increased, it was possible to verify that the workability was decreasing, therefore this is also related to the compaction strength, as reported in study by Akinyele and Ajede [23]. The compaction curves of the mixtures are presented in Figure 4. Moreover, the curves proved (especially between 0% and 30%) the difference between the mixtures in relation to the addition of waste, i.e., the higher the percentage of addition, the greater the difficulty of reaching the optimum moisture content with increasing percentage of PET.

#### 3.1.4. Microstructural Characterization

**Figure 5:**
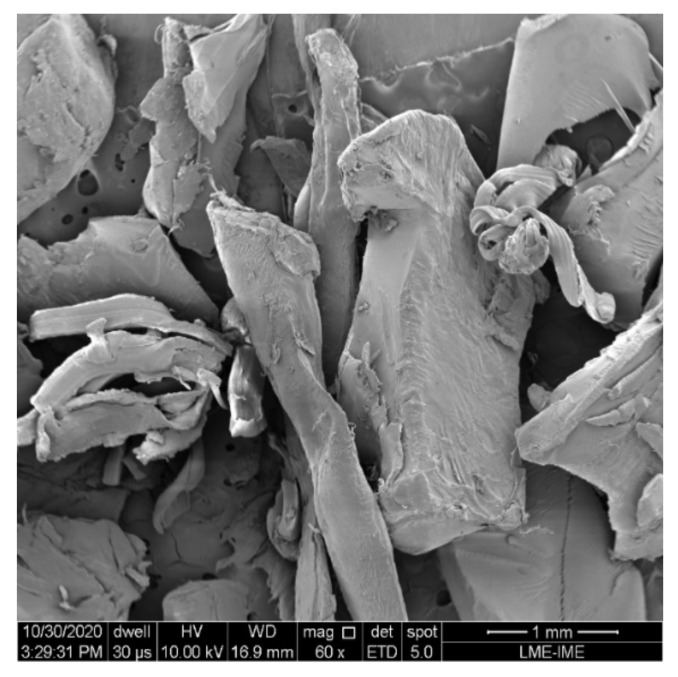
Scanning Electron Microscopy (SEM) of PET 60× magnification.

A morphological analysis in Figure 5 by SEM showed that PET presents high surface area and irregularities in the shape and size of its particles. These are typical of different cutting and crushing processes of the waste, as shown in studies by Siqueira and Holanda [49]. As the particles present an irregular distribution, smaller sized particles can occupy the free spaces left by larger sized particles, forming particle packing [50].

### 3.2. Technological Characterization of Bricks

#### 3.2.1. Compressive Strength

The compressive strength analysis of the bricks produced with different levels of in-corporation of PET waste showed a significant difference between the percentages, as well as between the ages. At seven days, the bricks made with 20% and 30% addition of PET waste showed the highest values of compressive strength, values that did not differ statistically between them. At 28 days, the highest average value was observed for the 20% addition (1.80 MPa), followed by 30% (1.45 MPa) and then the lowest values (0% and 10%) with no differences between them, as shown in Table 4.

According to the results, it was possible to observe that an increase in compressive strength occurred in bricks with different PET mixtures (20% and 30%). The samples with 20% PET reached the average value of compressive strength required by the ABNT standard NBR 15270-1 [51], which recommends 1.5 MPa for the total average of the samples. The mixtures with more PET had more water content. Since the PET particles were larger and the material was not porous, more water was available for cement hydration, which was probably the main reason why higher strengths were achieved, considering that the cementitious matrix does not have as good adhesion with PET [23].

The higher the concentration with smaller diameters of PET particles, the lower the probability of an increase in the number of voids and the direct interference in the strength of bricks [7]. In the case of 30% PET samples, Akinyele and Ajede [23] stated that the greater the amount of plastic, the greater the amount of water needed to improve workability; in this case, it is possible to further state that the lack of water needed for hydration also correlated with lower strength. However, the authors state that this waste can be considered to replace fine aggregates up to 20% in cement-based materials. Additionally, the worse workability of the mixtures with a high amount of PET could be attributed not only to the higher particle size, but also to the rough and irregular shapes of the PET particles, as shown in the SEM images. 

Regarding the reference sample (0% PET) and the samples of bricks with different incorporations of PET, they did not reach the average established by [43]. It is also estimated that they may have suffered interference in relation to cement hydration due to the type of cure used (humid chamber), damaging their strength. The incorrect hydration of the cement is generally due to the lack of water or humidity necessary for the cement to react, as shown in [49,50,51,52]. As for the ages, there was no difference for most of the treatments due to the cement used being CPV, which has greater strength gain at early ages in view of its better quality due to finer grinding. 

#### 3.2.2. Water Absorption

The analysis of water absorption in the bricks in Table 5 showed that there was no significant difference between the mixtures (*p* < 0.05). Thus, it was found that the amount of PET incorporated in all mixtures did not interfere with water absorption. 

According to the ABNT standard [43], the water absorption values should not present average values higher than 20%, nor individual values higher than 22%. Considering that PET is a material that does not absorb water, it is believed that it contributed to the bricks not presenting high water absorption. This was confirmed by Górak et al. [53] in studies of the effect of incorporating PET waste into cementitious composites, which indicated that the particle size of the waste has a significant effect on water absorption in relation to its porosity [54,55,56,57].

## 4. Conclusions

The soil used in the study, according to its particle distribution and sand characteristics, is considered suitable for use in the manufacturing of soil-cement bricks. Indeed, the soil with 57.1% sand fraction, 24.3% clay fraction and 18.6% silt fraction is classified as sandy clay soil. The PET waste corresponded to 99.9% of sandy particle fraction, with 85.5% coarse particles, 11.5% medium particles and 2.9% fine particles. It was possible to verify that as the addition of PET increased in the mixtures, the higher the content of sandy particles.

As for the optimal moisture content and compaction energy, it was observed that the natural soil (without waste addition) showed optimal moisture value and maximum specific weight satisfactory. For the mixtures, the greater the addition of PET (10%, 20% and 30%), the greater the optimum humidity of compaction, in addition to a decrease in maximum specific weight. This revealed that the higher the addition of water, the lower the workability of the mixture, thus interfering in the mechanical strength.

The evaluated PET waste can be classified as sandy grain size and non-plastic waste. Through its microstructure it was possible to verify the rough and irregular shapes of the PET due to its crushing and processing. 

Based on the evaluation of the physical and mechanical tests of soil-cement bricks, it was possible to verify improvement in their properties with the incorporation of PET. The average compressive strength with the incorporation of 20% PET managed to reach the value of 1.80 MPa, i.e., above 1.50 MPa which is the minimum value established by the Brazilian standard. As for water absorption, the bricks showed satisfactory values and complied with the values established by the standard, i.e., not presenting average values higher than 20% or individual values higher than 22%.

For future studies related to the results of this work, it would be important to consider that although the amount of water in the mixture increased according to the amount of PET, it could compensate with an additional hydration of cement particles, suggesting higher strengths of the bricks.

Thus, the incorporation of PET in soil-cement bricks can be considered an alternative for non-structural applications, such as closing walls in building construction. Moreover, this study verifies that it is possible to reduce the environmental impacts of this type of waste, as demonstrated in the bricks made with 20% PET waste. However, it is necessary to consider further studies regarding the life cycle of this type of material, especially its final cycle and durability, in order to enhance applications and avoid greater environmental impacts.

## Figures and Tables

**Table 1:** Compositions (vol.%) of the soil-cement mixtures.

Mixtures	Soil (vol.%)	Cement (vol.%)	PET (vol.%)
0%	90	10	0
10%	80	10	10
20%	70	10	20
30%	60	10	30

**Table 2:** Sample consistency limits.

PET (%)	LL (%)	PL (%)	PI (%)
0%	40.1	22.4	17.7
10%	34.0	20.0	14.0
20%	36.7	19.4	17.3
30%	33.8	18.4	15.3

* Liquidity Limit (LL), Plasticity Limit (PL) and Plasticity Index (PI).

**Table 3:** Optimum compaction humidity and maximum mixture densities.

PET	Specific Weight (kN/m^3^)	Optimum Humidity (%)
0%	17.8	14.5
10%	16.6	15.0
20%	15.6	15.3
30%	13.9	16.5

**Table 4:** Compressive strength of samples at 7 and 28 days.

PET (%)	Compressive Strength 7 Days	Standard Deviation 7 Days	Compressive Strength 28 Days	Standard Deviation 28 Days
0	0.68 Ba	0.132	0.83 Ca	0.115
10	0.88 Ba	0.053	0.89 Ca	0.043
20	1.56 Ab	0.270	1.80 Aa	0.111
30	1.39 Aa	0.120	1.45 Ba	0.118

* Val. * Values followed by the same letter, uppercase in the column and lowercase in the row, do not differ at the level of 5% by Tukey’s test.

**Table 5:** Water absorption of samples at 28 days.

PET (%)	Water Absorption	Standard Deviation
0	16.23 A	1.057
10	15.93 A	0.109
20	15.27 A	0.252
30	15.21 A	0.283

* Values followed by the same letter, uppercase letters in the column do not differ at the 5% level by Tukey’s test.
